# Attention to blood pressure cuff sizes is important for home and in-clinic blood pressure measurement

**DOI:** 10.1038/s41440-025-02308-7

**Published:** 2025-08-21

**Authors:** Ritu Trivedi, Niamh Chapman, Shiva R. Mishra, Mark R. Nelson, Clara K. Chow, Dean S. Picone

**Affiliations:** 1 School of Health Sciences, Faculty of Medicine and Health, The University of Sydney, Sydney, NSW Australia; 2 Menzies Institute for Medical Research, University of Tasmania, Hobart, TAS Australia; 3 Population Health Research & Epidemiology, South Western Sydney Local Health District, Liverpool, NSW Australia; 4 Population Health, School of Medicine, Western Sydney University, Sydney, NSW Australia; 5 Westmead Applied Research Centre, Faculty of Medicine and Health, The University of Sydney, Sydney, NSW Australia; 6 Cardiology department, Westmead Hospital, Westmead, NSW Australia

**Keywords:** Hypertension, Blood pressure, Blood pressure determination, Blood pressure monitors, Implemental hypertension

## Abstract

Arm circumference determines appropriate blood pressure cuff size, which is critical for accurate measurements. This cross-sectional analysis aimed to assess cuff size needs according to mid-arm circumferences of Australian adults. Based on typical in-clinic cuff sizes, most Australians would require a medium (51.7%) or large (44.5%) cuff, which means that the cuff would need to be changed for almost every second patient. Most home devices are supplied with a standard (22–32 cm) or wide-range (22–42 cm) cuff, these were found to be unsuitable for 9 million adults (48.3%) and over 701,995 adults (3.8%), respectively. Concerningly, the standard cuff size available with commonly used home devices would be unsuitable for large proportions of people in higher cardiovascular risk groups (e.g. hypertension (59.7%), diabetes (66.3%), high cholesterol (55.6%) and obesity (92.3%)). This work highlights attention must be paid to selecting appropriate cuff sizes for accurate blood pressure measurements in-clinic and at home.

**Figure:**
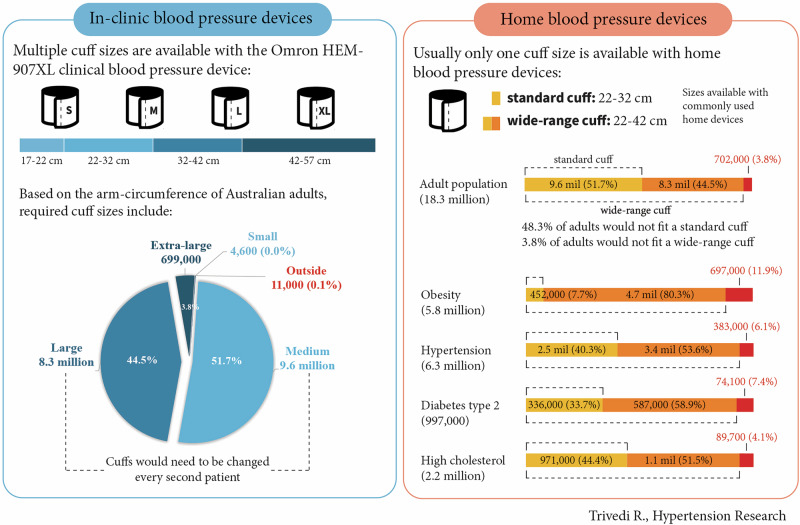
Arm circumference of Australian adults to assess cuff size requirements for in-clinic and home blood pressure measurements.

Arm circumference of Australian adults to assess cuff size requirements for in-clinic and home blood pressure measurements.

## Abstract (web summary)

Appropriate blood pressure cuff size is critical for accurate measurements. Most Australians require a medium (51.7%) or large (44.5%) cuff meaning the cuff needs to be changed for about every second patient. Yet most home devices are supplied with one cuff size which is not suited to over 700,000 adults.

## Introduction

Hypertension is the leading cause of preventable cardiovascular disease and mortality [1] affecting 1.4 billion people worldwide [2], including one-in-three Australians [3]. Adequate blood pressure (BP) control is essential in preventing stroke, heart failure and dementia, but poor control rates persist to be a problem globally [2]. Optimal diagnosis and management are essential in improving control rates but rely on obtaining accurate BP measurements to inform clinical management decisions.

To achieve accurate BP measurement, using a validated device with an appropriately sized cuff is crucial for measurements taken both in-clinic and in home settings [4]. Most in-clinic devices are equipped with a variety of cuff sizes to fit different arms. In the clinic, a cuff may already be connected to the monitor but need to be changed to another size for different patients, but this may not always be done [5]. On the other hand, home devices are typically sold with a single cuff size, the most common being 22–32 cm (standard) and 22–42 cm (wide-range) cuffs [6]. Because clinical guidelines recommend both in-clinic and home BP monitoring, choosing the correct cuff size is important in both these settings to enable accurate BP measurements so that appropriate clinical decisions can be made. Therefore, the aim of this study was to assess the need for different BP cuff sizes, based on the population distribution of adult arm circumferences in Australia.

## Methods

This paper reports on a cross-sectional analysis of mid-arm circumference (MAC) among Australian adults to assess appropriateness of cuff sizes of common in-clinic and home BP devices. Nationally representative population characteristics were obtained from the National Health Survey 2017-18 [7]. This data was used to extract weight and estimate MAC from a published equation [MAC = (weight+50)/4] [8] due to the lack of nationally representative data on arm circumferences of Australian adults.

Two separate analyses were conducted. Firstly, MAC was categorised by cuff sizes available with a commonly used in-clinic device, the Omron HEM-907XL which comes with four separate cuffs: small (17–22 cm), medium (22–32 cm), large (32–42 cm), extra-large (42–57 cm). It was chosen as the in-clinic device for this analysis as a large number ( ~ 20,000 units) of the Omron HEM-907 devices were distributed by the High Blood Pressure Research Council of Australia to physicians in primary care in 2006/7 [9]. The Omron HEM-907XL is an updated version of the HEM-907 that was upgraded to allow inflation and measurement using an extra-large cuff. It was included in this analysis due to its use in major trials such as SPRINT [10]. Secondly, MAC was categorised by cuff sizes available with popular home devices: standard (22–32 cm) or wide-range (22–42 cm) cuffs. This assumption was based on a recent national survey indicating the top ten most commonly used home devices (3 included a standard cuff size) [6].

Descriptive analyses were stratified by sex, age, body mass index and history of cardiometabolic risk factors (hypertension, type II diabetes, high cholesterol). Hypertension was defined as people who had measured high BP ( ≥ 140/90 mmHg), or normal measured BP and were taking antihypertensive medications [7].

## Results

The mean estimated MAC of 18.7 million Australian adults was 32.3 ± 4.8 cm. Among this group, cuff sizes required for the in-clinic device were: 0.0% (4600) small, 51.7% (9.6 million) medium, 44.5% (8.3 million) large and 3.7% (700,000) extra-large (Table 1). A small proportion 0.1% (11,000) fell outside these sizes. Whereas, for home devices, the standard 22-32 cm cuff would not fit 48.3% (9 million) adults and the wide-range 22–42 cm cuff would not fit 3.8% (701,995) adults (Table 2). The proportions with unsuitable cuff fit were greater among high cardiovascular risk populations including those with obesity (standard: 92.3% and wide-range: 11.9%), hypertension (standard: 59.7% and wide-range: 6.1%), diabetes type II (standard: 66.3% and wide-range: 7.4%) and high cholesterol (standard: 55.6% and wide-range: 4.1%).

**Table 1:** Arm circumference of Australian adults categorised by cuff sizes available with commonly used in-clinic device Omron HEM-907XL

	Total population^a^		Cuff sizes available with commonly used in-clinic device: Omron HEM-907XL									
	*N*	%	Small (17 to <22 cm)		Medium (22 to <32 cm)		Large (32 to <42 cm)		Extra large (42 to ≤57 cm)		Outside	
			*N*	%	*N*	%	*N*	%	*N*	%	*N*	%
**Total**	18,655,100	100%	4,606	0.0%	9,643,914	51.7%	8,296,541	44.5%	698,658	3.7%	11,381	0.1%
**Arm circumference**^**b**^**, cm**												
Mean (SD)	32.3 (4.82)		20.6 (0.61)		28.7 (2.13)		35.4 (2.49)		45.2 (2.98)		59.4 (1.59)	
**Sex**												
Women	9,511,803	51.0%	4606	0.0%	6,606,194	69.5%	2,710,285	28.5%	188,596	2.0%	2123	0.0%
Men	9,143,297	49.0%	0	0.0%	3,037,720	33.2%	5,586,256	61.1%	510,062	5.6%	9258	0.1%
**Age, years**												
18–29	3,900,634	20.9%	2975	0.1%	2,378,157	61.0%	1,390,333	35.6%	129,169	3.3%	0	0.0%
30–39	3,632,991	19.5%	0	0.0%	1,908,700	52.5%	1,544,024	42.5%	171,367	4.7%	8900	0.2%
40–49	3,143,838	16.9%	0	0.0%	1,485,651	47.3%	1,532,160	48.7%	123,546	3.9%	2481	0.1%
50–59	2,976,838	16.0%	0	0.0%	1,341,882	45.1%	1,496,278	50.3%	138,678	4.7%	0	0.0%
60–69	2,518,625	13.5%	249	0.0%	1,112,386	44.2%	1,309,743	52.0%	96,248	3.8%	0	0.0%
70–79	1,699,849	9.1%	0	0.0%	885,343	52.1%	778,066	45.8%	36,440	2.1%	0	0.0%
80 and over	782,326	4.2%	1382	0.2%	531,795	68.0%	245,938	31.4%	3211	0.4%	0	0.0%
**Body mass index**^**c**^**, kgm**^**-2**^												
Underweight	243,593	1.3%	1631	0.7%	241,962	99.3%	0	0.0%	0	0.0%	0	0.0%
Normal weight	5,920,281	31.7%	2975	0.1%	5,562,098	93.9%	355,209	6.0%	0	0.0%	0	0.0%
Overweight	6,646,936	35.6%	0	0.0%	3,387,975	51.0%	3,258,275	49.0%	686	0.0%	0	0.0%
Obese	5,844,290	31.3%	0	0.0%	451,879	7.7%	4,683,058	80.1%	697,972	11.9%	11,381	0.2%
**Conditions**												
Hypertension^**d**^	6,280,113	33.7%	1382	0.0%	2,532,168	40.3%	3,351,338	53.4%	385,980	6.1%	9245	0.1%
Diabetes (type II)^e^	997,209	5.3%	249	0.0%	335,973	33.7%	582,023	58.4%	78,965	7.9%	0	0.0%
High cholesterol^e^	2,188,867	11.7%	512	0.0%	971,293	44.4%	1,124,870	51.4%	88,245	4.0%	3947	0.2%

^a^Population characteristics from the Australian National Health Survey 2017-18 [7]. ^b^Mid-arm circumference (MAC) was estimated from a previously published equation [MAC = (weight + 50)/4] [8]. ^c^Body mass index categories: <18.5 kgm^-2^ (underweight), ≥18.5 to <25.0 kgm^-2^ (normal weight), ≥25.0 to <30.0 kgm^-2^ (overweight) and ≥30.0 kgm^-2^ (obese). ^d^Hypertension flag captures those with measured high blood pressure, or normal measured blood pressure and taking medications [7]. ^e^Self-reported [7]

**Table 2:** Arm circumference of Australian adults categorised by cuff sizes available with commonly used home blood pressure devices

	Total population^a^		Cuff sizes available with common home blood pressure devices							
	*N*	%			Wide-range (22 to ≤42 cm)					
			Outside ( < 22 cm)		Standard (22 to <32 cm)		32 to ≤42 cm		Outside ( > 42 cm)	
			*N*	%	*N*	%	*N*	%	*N*	%
**Total**	18,655,100	100%	4606	0.0%	9,643,914	51.7%	8,309,192	44.5%	697,389	3.7%
**Arm circumference**^**b**^**, cm**										
Mean (SD)	32.3 (4.82)		20.6 (0.61)		28.7 (2.13)		35.4 (2.50)		45.4 (3.46)	
**Sex**										
Women	9,511,803	51.0%	4606	0.0%	6,606,194	69.5%	2,712,700	28.5%	188,304	2.0%
Men	9,143,297	49.0%	0	0.0%	3,037,720	33.2%	5,596,492	61.2%	509,085	5.6%
**Age, years**										
18-29	3,900,634	20.9%	2975	0.1%	2,378,157	61.0%	1,390,333	35.6%	129,169	3.3%
30-39	3,632,991	19.5%	0	0.0%	1,908,700	52.5%	1,548,018	42.6%	176,273	4.9%
40-49	3,143,838	16.9%	0	0.0%	1,485,651	47.3%	1,532,433	48.7%	125,753	4.0%
50-59	2,976,838	16.0%	0	0.0%	1,341,882	45.1%	1,503,289	50.5%	131,667	4.4%
60-69	2,518,625	13.5%	249	0.0%	1,112,386	44.2%	1,310,428	52.0%	95,562	3.8%
70-79	1,699,849	9.1%	0	0.0%	885,343	52.1%	778,752	45.8%	35,754	2.1%
80 and over	782,326	4.2%	1382	0.2%	531,795	68.0%	245,938	31.4%	3211	0.4%
**Body mass index**^**c**^**, kgm**^**-2**^										
Underweight	243,593	1.3%	1631	0.7%	241,962	99.3%	0	0.0%	0	0.0%
Normal weight	5,920,281	31.7%	2975	0.1%	5,562,098	93.9%	355,209	6.0%	0	0.0%
Overweight	6,646,936	35.6%	0	0.0%	3,387,975	51.0%	3,258,961	49.0%	0	0.0%
Obese	5,844,290	31.3%	0	0.0%	451,879	7.7%	4,695,023	80.3%	697,389	11.9%
**Conditions**										
Hypertension^d^	6,280,113	33.7%	1382	0.0%	2,532,168	40.3%	3,363,177	53.6%	383,386	6.1%
Diabetes (type II)^e^	997,209	5.3%	249	0.0%	335,973	33.7%	587,139	58.9%	73,849	7.4%
High cholesterol^e^	2,188,867	11.7%	512	0.0%	971,293	44.4%	1,127,915	51.5%	89,147	4.1%

One cuff size is generally available with home blood pressure devices, according to the survey by Clapham et al. 2025, the 22-32 cm (standard) and 22–42 cm (wide-range) cuff is accompanied with commonly used home devices [6]. ^a^Population characteristics from the Australian National Health Survey 2017-18 [7]. ^b^Mid-arm circumference (MAC) was estimated from a previously published equation [MAC = (weight + 50)/4] [8]. ^c^Body mass index categories: <18.5 kgm^-2^ (underweight), ≥18.5 to <25.0 kgm^-2^ (normal weight), ≥25.0 to <30.0 kgm^-2^ (overweight) and ≥30.0 kgm^-2^ (obese). ^d^Hypertension flag captures those with measured high blood pressure, or normal measured blood pressure and taking medications [7]. ^e^Self-reported [7]

## Discussion

A key novel finding of this analysis was that cuff sizes provided with in-clinic devices need to be changed for every second Australian adult because most require either a medium (51.7%) or large (44.5%) cuff size. Additionally, home devices, which are usually supplied with a single cuff size, are unlikely to fit at least 700,000 Australians but could be supplied with an inappropriate cuff size for up to 9 million Australians.

Using the correct cuff size is important for accurate BP measurements and could have significant implications for the management of hypertension. Data from a randomised crossover trial found that a cuff which was one size too small can overestimate systolic BP by 4.8 mmHg, whereas a cuff that was one size too large can underestimate by −3.6 mmHg [4]. These errors could influence population level estimates of BP, hypertension prevalence and have wide-spread clinical implications for individuals given that a 5-mmHg error in measurement could lead to misclassification of hypertension status in 84 million individuals worldwide [11].

In-clinic devices are equipped with multiple cuff sizes, meaning cuffs need to be swapped to ensure appropriate fit to the patient’s arm. Based on our study, the medium and large cuff sizes would be the most commonly used in clinical settings which is similar to the findings reported in the United States [12]. However, there is evidence suggesting physicians and nurses have suboptimal knowledge about in-clinic BP methods, including cuff size [5]. Using an incorrect cuff size was among the common errors that occurred during observations of in-clinic BP assessments performed by nurses and medical assistants in primary care [5]. Moreover, if medical practices in Australia are still using the Omron HEM-907 device, it is possible that the extra-large cuff size may not be available because that size cuff only became available with the Omron HEM-907XL device (FDA clearance obtained 11.02.2004). A potential consequence is that a large sized cuff might be used for 3.7% of the Australian adult population who instead need an extra-large cuff, which would lead to overestimation of BP [4]. Moreover, in clinical practice swapping cuffs requires time and consideration of where to store different cuff sizes to help facilitate easy integration of using the correct cuff size into clinical workflows. The use of inappropriate cuff sizes, along with other variations in measurement technique and time pressures in health care settings, is likely to cause high variability in BP measured in-clinic which can adversely impact the quality and clinical utility of the measurements. Addressing each of these factors could improve measurement quality and hypertension management.

Home BP devices are usually only supplied with a single cuff size. A recent survey found that three of the top ten most popular devices used for home BP measurement in Australia were only supplied with a standard 22–32 cm cuff size [6]. Based on our results, a standard cuff size would be unsuitable for 48% of all adults, but importantly 60% with hypertension. As obesity rates continue to rise, the proportion of people requiring extra-large cuffs ( > 42 cm) is also expected to increase [12]. For those with arm circumferences >42 cm, some clinical guidelines suggest the use of validated wrist-cuff devices or conical upper-arm cuffs, although the latter are not widely available [13]. It is critical that businesses selling home BP devices, such as pharmacies, support people to purchase validated devices that have a correctly sized cuff for each person. Some pharmacies stock generic extra-large cuffs that are marketed as compatible with many devices. But these should not be used because all automatic devices are validated for accuracy with the specific cuffs supplied meaning use of generic cuffs could result in unreliable BP measurements. Moreover, as people shift to purchasing of BP devices online, clear reporting of validation, suitability to population and cuff size should also be prioritised. Raising awareness among the general community and health professionals on the importance of selecting a validated device with the correctly sized cuff is key to ensuring accurate, reliable measurements to inform clinical decisions and optimise self-management.

The primary limitation was that MAC was estimated due to lack of representative, measured MAC data for Australia. The MAC was estimated using weight in the calculation and the equation was based on modelling conducted in a cohort in the United States with a marginally higher average weight than reported in the Australian population [8]. A potential consequence of using an estimated MAC is underestimation of the proportion of Australian adults with a MAC between 17 and 22 cm (small cuff size) who may be frail or from underrepresented groups. It is important that this population, like all others, also have a correctly sized cuff used for BP measurement and this may particularly be relevant for home devices, which are commonly only supplied with a 22–42 cm cuff size. Overall, there is a need for nationally representative data for measured MAC from various populations (e.g., different ages, ethnicities, medical conditions) to understand cuff size requirements and ensure accurate measurements of BP. Nevertheless, the observations are plausible and in-line with recent findings from similar countries [12]. Moreover, the current study reports arm circumference specific to an Australian population and distributions may differ according to countries, regions and populations. The need to use appropriate cuff sizes for BP measurement is globally relevant and important for optimal hypertension management to improve BP control rates globally.

In conclusion, arm circumferences span multiple BP cuff sizes available with in-clinic and home BP devices, reiterating the need to pay close attention to cuff sizes to achieve accurate BP measurement. Future research should determine whether cuff sizes are being changed in-clinic, and the barriers or enablers to doing this. Additionally, exploring whether people are purchasing home monitors with cuffs that suit their arm circumferences is also required.

## References

[1] Murray CJ, Aravkin AY, Zheng P, Abbafati C, Abbas KM, Abbasi-Kangevari M, et al. Global burden of 87 risk factors in 204 countries and territories, 1990–2019: a systematic analysis for the Global Burden of Disease Study 2019. Lancet. 2020;396:1223–49. 33069327 10.1016/S0140-6736(20)30752-2 PMC7566194

[2] Mills KT, Stefanescu A, He J. The global epidemiology of hypertension. Nat Rev Nephrol. 2020;16:223–37. 32024986 10.1038/s41581-019-0244-2 PMC7998524

[3] AIHW. Heart, stroke and vascular disease: Australian facts. Canberra: Australian Institute of Health and Welfare; 2024.

[4] Ishigami J, Charleston J, Miller ER, Matsushita K, Appel LJ, Brady TM. Effects of cuff size on the accuracy of blood pressure readings: the Cuff (SZ) randomized crossover trial. JAMA Intern Med. 2023;183:1061–8. 37548984 10.1001/jamainternmed.2023.3264 PMC10407761

[5] Todkar S, Padwal R, Michaud A, Cloutier L. Knowledge, perception and practice of health professionals regarding blood pressure measurement methods: a scoping review. J Hypertens. 2021;39:391–9. 33031184 10.1097/HJH.0000000000002663

[6] Clapham E, Carmichael S, Picone DS, Schutte AE, Slater K, Stevens J, et al. How and why do Australians obtain blood pressure devices for use at home? A mixed-methods study. 2025. Preprint available at https://www.medrxiv.org/content/10.1101/2025.02.27.24318446v1.

[7] ABS. National Health Survey: First Results methodology. *Australian Bureau of Statistics*; 2017–18.

[8] Cattermole GN, Graham CA, Rainer TH. Mid-arm circumference can be used to estimate weight of adult and adolescent patients. Emerg Med J. 2017;34:231–6. 27993936 10.1136/emermed-2015-205623 PMC5502250

[9] Myers MG, Nelson MR, Head GA. Automated office blood pressure measurement for routine clinical practice. Med J Aust. 2012;197:372–3. 23025727 10.5694/mja11.11545

[10] SPRINT Research Group. A randomized trial of intensive versus standard blood-pressure control. N Engl J Med. 2015;373:2103–16. 26551272 10.1056/NEJMoa1511939 PMC4689591

[11] Padwal R, Campbell NR, Schutte AE, Olsen MH, Delles C, Etyang A, et al. Optimizing observer performance of clinic blood pressure measurement: a position statement from the Lancet Commission on Hypertension Group. J Hypertens. 2019;37:1737–45. 31034450 10.1097/HJH.0000000000002112 PMC6686964

[12] Jackson SL, Gillespie C, Shimbo D, Rakotz M, Wall HK. Blood pressure cuff sizes for adults in the United States: National Health and Nutrition Examination Survey, 2015–2020. Am J Hypertens. 2022;35:923–8. 36066190 10.1093/ajh/hpac104 PMC10851131

[13] Mancia G, Kreutz R, Brunström M, Burnier M, Grassi G, Januszewicz A, et al. 2023 ESH Guidelines for the management of arterial hypertension The Task Force for the management of arterial hypertension of the European Society of Hypertension: Endorsed by the International Society of Hypertension (ISH) and the European Renal Association (ERA). J Hypertens. 2023;41:1874–2071. 37345492 10.1097/HJH.0000000000003480

